# Treatment Sequences in Patients with Metastatic Colorectal Cancer in Japan: Real-World Evidence of First- to Fifth-Line Treatments

**DOI:** 10.3390/cancers17243962

**Published:** 2025-12-12

**Authors:** Yoshinori Kagawa, Tsuyoshi Osaka, Toshiki Imamura, Hiroyo Kuwabara

**Affiliations:** 1 Department of Gastroenterological Surgery, Osaka International Cancer Institute, Osaka 541-8567, Japan; 2 Japan Medical Affairs, Japan Oncology Business Unit, Takeda Pharmaceutical Company Limited, Tokyo 103-8668, Japan; tsuyoshi.osaka@takeda.com (T.O.); toshiki.imamura@takeda.com (T.I.); hiroyo.kuwabara@takeda.com (H.K.)

**Keywords:** metastatic colorectal cancer, real world, treatment sequence, transition rate, systemic chemotherapy, later-line treatment

## Abstract

**Background:** The development of later-line drugs for metastatic colorectal cancer (mCRC) has expanded treatment options. However, real-world evidence of treatment sequences and transition rates from early- to later-line treatments are limited. **Patients and methods:** This was a retrospective study using hospital administrative data from patients in Japan who underwent colorectal cancer surgery or first-line treatment after January 2017. Transition rates were calculated and treatment sequences were summarized using a Sankey diagram. Logistic regression was performed to identify factors associated with the transition from second- to third-line treatment. **Results:** In total, 27,100 patients (median age: 69 years) were included in the study population. Transition rates to subsequent treatment lines from first to fifth ranged from 66.6% to 71.3% (first to second: 69.4%; second to third: 71.3%; third to fourth: 71.1%; fourth to fifth: 66.6%). Among 9061 patients who received second-line treatment, 6456 continued to third-line treatment, and 2605 received the best supportive care. Longer first- (≥180 days; OR: 1.24; 95% CI: 1.13–1.37) and second-line (≥120 days; OR: 1.70; 95% CI: 1.55–1.86) treatment durations were significant factors for continuing to third-line treatment. Prior therapy with oxaliplatin and irinotecan plus molecular targeted drugs was also associated with a higher likelihood of proceeding to third-line treatment (OR: 1.41; 95% CI: 1.27–1.56). **Conclusions:** This study describes the current mCRC treatment landscape in Japan. Considering the findings, appropriate early treatments are critical for transition to later-line treatment. Additionally, many later-line options are necessary to provide treatment continuation opportunities for better outcomes.

## Simple Summary

This study describes treatment sequences of systemic chemotherapy for patients with metastatic colorectal cancer in a real-world setting in Japan. Each transition rate from first- to second-, second- to third-, third- to fourth-, and fourth- to fifth-line treatment was approximately 70%. Compared with patients who transitioned to the best supportive care, patients who transitioned to third-line treatments received first-and second-line treatments for longer and were more likely to have been prescribed regimens including oxaliplatin and irinotecan-based chemotherapy plus vascular endothelial growth factor and/or epidermal growth factor receptor inhibitors in early-line treatments.

## 1. Introduction

Colorectal cancer (CRC) ranks as the third most common cancer and the second leading cause of cancer-related death around the globe [1]. Among patients with newly diagnosed CRC, 20% present with metastatic disease at the time of diagnosis, while an additional 25% of those initially diagnosed with localized disease subsequently develop metastases [2].

The primary objective of systemic chemotherapy in treating patients with unresectable metastatic CRC (mCRC) is to prolong overall survival (OS) while maintaining quality of life. Over the past two decades, monoclonal antibodies targeting vascular endothelial growth factor (VEGF) and epidermal growth factor receptor (EGFR) were introduced as early-line treatments, followed by regorafenib (REG) and trifluridine/tipiracil (FTD/TPI) as later-line treatments for patients with mCRC. Furthermore, fruquintinib (FRU) recently demonstrated efficacy and safety in the FRESCO-2 study [3] and received regulatory approval as a later-line treatment option in many countries and regions, including the USA, the EU, and Japan. The combination and sequential use of these newly developed and conventionally used cytotoxic drugs have significantly improved median OS in patients with mCRC; recent randomized clinical trials show an OS of over 30 months in the first-line setting [4,5,6,7,8]. Alongside these newer drugs and results, international guidelines from the American Society of Clinical Oncology (ASCO) [9], European Society of Medical Oncology (ESMO) [10], and Japanese Society for Cancer of the Colon and Rectum (JSCCR) [11] have been updated accordingly and recommend treatment sequences from early to late lines of treatment. However, it is not always feasible for patients with mCRC to adhere to the established guidelines in real-world settings, owing to the presence of comorbidities, complications, and other factors, particularly in the later stages of treatment. Furthermore, patients with CRC are often elderly; approximately 56% of patients were over 65 years of age in a previous report [12]. The treatment status of patients with mCRC in real-world settings has not been well explored.

To optimize strategies for the treatment of patients with mCRC in practical scenarios, it is important to understand treatment sequences of systemic chemotherapy from early to later lines in daily practice. Although several studies have investigated the sequences of mCRC, these studies have primarily focused on specific first- or second-line regimens [13,14,15,16,17]. Although some research has investigated treatments beyond the third line, this has typically focused on specific therapies or subsets of patients with mCRC [18,19,20]. The development of later-line drugs since the 2010s has expanded treatment options; however, studies describing treatment sequences from early to later lines in the mCRC population are limited [21], and optimal strategies across the entirety of treatment sequences are unclear.

The objective of this study was to describe the treatment sequences and transition rates of patients with mCRC in Japanese real-world practice, with a particular focus on patients who reached third-line therapy and beyond. Furthermore, we aimed to identify the factors associated with patients’ continuation from second-line to third-line chemotherapy.

## 2. Materials and Methods

### 2.1. Study Design and Data Source

This was an observational study using a large hospital-based administrative claims database constructed by Medical Data Vision Co. Ltd. (MDV) in Japan [22,23], which comprised approximately 44.1 million cumulative patient records from 500 acute hospitals, including 245 cancer-designated hospitals, in Japan at the time this study was conducted. The records included demographic (such as age and sex) and inpatient and outpatient claim information. All data were anonymized; informed consent and ethical committee approval were waived because the Japanese Ethical Guidelines for Medical and Health Research Involving Human Subjects do not apply to research focusing solely on de-identified data.

### 2.2. Study Population

**Figure 1:**
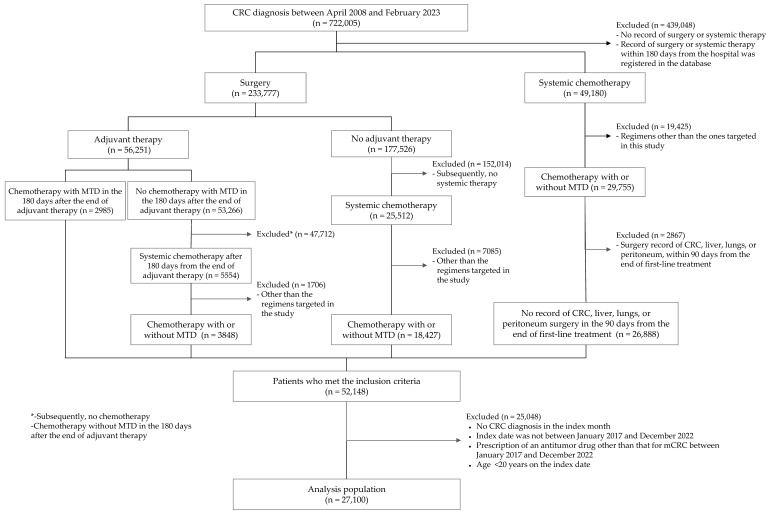
Patient disposition. MTD, molecular targeted drug.

The study population was identified in accordance with methodologies employed in previous studies [13]. To ascertain the mCRC population, patients with a diagnosis of CRC (International Classification of Diseases, 10th edition code: C18–C20) during the study period (1 April 2008, to 28 February 2023) were initially extracted from the MDV database and then classified into two groups: those who initiated treatment with CRC surgery and those who initiated treatment with systemic chemotherapy (Figure 1). Subsequently, to create an analysis data set that excluded neoadjuvant chemotherapy and other pertinent factors, the extracted population was divided into following three cohorts: the early recurrence cohort (ERC), the molecular targeted therapy cohort (MTC), and the nonmolecular targeted therapy cohort (non-MTC). The ERC included patients who began adjuvant chemotherapy in the 90 days after surgery and subsequently started a regimen containing a molecular targeted drug such as bevacizumab (BEV), cetuximab (CET), or panitumumab (PANI) in the 180 days after completing adjuvant chemotherapy. The MTC included patients who started molecular targeted chemotherapy as their first-line treatment either directly, after CRC surgery, or more than 180 days after surgery and adjuvant chemotherapy. Patients with records of CRC, liver, or lung resection or peritoneal surgery in the 90 days after completing first-line treatment were excluded, because this indicated neoadjuvant therapy. The non-MTC was identified in a similar manner to the MTC but included patients starting first-line oxaliplatin (OX)- or irinotecan (IRI)-based chemotherapy without molecular targeted agents; this cohort was defined as a part of the study population to reflect real-world patients unable to start their first-line chemotherapy with molecular targeted drugs.

The index date was defined as the date of CRC resection surgery for the ERC and the start date of first-line treatment for both the MTC and the non-MTC. Patients were excluded if they met any of the following criteria: those whose index date did not fall between 1 January 2017, and 31 December 2022, to focus on recent late-line practice in real-world conditions; those who had prescription records for antitumor drugs other than those for CRC within this period; those who did not have a diagnosis of CRC in the month of their index date; and those who were under 20 years of age on the index date.

### 2.3. Treatment Regimens and Sequences

Treatment regimens for mCRC were defined based on prescription records for drugs considered standard treatment in the JSCCR treatment guidelines for colorectal cancer [11], and they were identified based on the combination of drugs prescribed in the 28 days after initiating each treatment line. Treatment was considered discontinued if none of the drugs in the regimen were prescribed for 180 days or more or if a new drug not originally in the regimen was prescribed after 28 days from the start of treatment. However, certain molecular targeted drugs (CET, PANI, BEV, ramucirumab [RAM], and aflibercept [AFL]) could be added to the regimen even after 29 days (add-on) [24]. The end date of a treatment line was determined by the earlier of the following: the date obtained by adding the number of days of treatment to the last prescription date of the regimen, or the day before the start date of the next treatment line. Censoring occurred when there were no visit records in the 60 days after the end date of a treatment. As for patients from the ERC, chemotherapy after adjuvant therapy was defined as second-line treatment, and adjuvant therapy was combined with first-line treatment from the MTC and the non-MTC.

To calculate transition rates, patients who were not censored were defined as with either ‘next line observed’ or ‘no next line observed’. Transition rates were then calculated as follows: next line observed/(all patients who received the line of treatment [next line observed + no next line observed]). Patients with ‘next line observed’ had subsequent treatment, and those with ‘no next line observed’ did not have records of any treatment regimens defined in this study but had records of a hospital visit in the 60 days after the end of the previous treatment; therefore, they were considered to have received the best standard of care (BSC).

### 2.4. Statistical Analysis

The analysis was conducted in two steps: first, we examined the overall study population, and we then evaluated the subpopulation of patients who received second-line treatment.

For the overall study population, each transition rate from first- to second-, second- to third-, third- to fourth-, and fourth- to fifth-line treatments was calculated. The distribution of regimens in patients who started each treatment line was summarized, and sequences from first- to fifth-line treatments were described using a Sankey diagram.

For the subpopulation of patients who received second-line treatment, the patient characteristics at the end of this treatment were summarized and stratified by those who continued to third-line treatment and those who transitioned to the BSC. Patient characteristics at the first line were also summarized for patients who started third-line treatment. Patient characteristics included demographics and patient background factors such as metastatic sites, comorbidities, the duration of treatment lines, etc. Descriptive statistics were recorded as medians and interquartile ranges (IQRs) for continuous variables and as numbers and percentages for categorical variables. A logistic regression model was developed to analyze factors associated with continuation to third-line treatment versus transition to the BSC, which was set as the dependent variable. Independent variables included in the model were age, sex, primary tumor location, metastasis (liver, lung, peritoneal, lymph node), duration of first-line and second-line treatments, and prior treatment with OX and IRI plus VEGF and/or EGFR inhibitors. The odds ratios (ORs) and corresponding 95% confidence intervals (CIs) were calculated for each independent variable. Finally, a Sankey diagram of the first through fifth treatment lines was created for this population to describe the treatment sequence. Durations of third-, fourth- and fifth-line treatments were also calculated, recorded using medians and IQRs. Data was processed using the Amazon Athena engine, version 3 (Amazon.com, Inc., Seattle, WA, USA), and statistical analyses were conducted using R, version 4.3.1 (The R Project for Statistical Computing).

## 3. Results

Patient disposition is shown in Figure 1. Of 722,005 patients with a CRC diagnosis recorded between 1 April 2008, and 28 February 2023, 27,100 were included in the overall study population after applying the inclusion and exclusion criteria (ERC: 1335 [4.9%]; MTC: 20,093 [74.1%]; non-MTC: 5672 [20.9%]). Median age was 69 years old (IQR: 61–75) and 61.3% of patients were men (Table S1).

### 3.1. Transition Rates and Early-Line Treatment Sequences in the Overall Population

**Figure 2:**
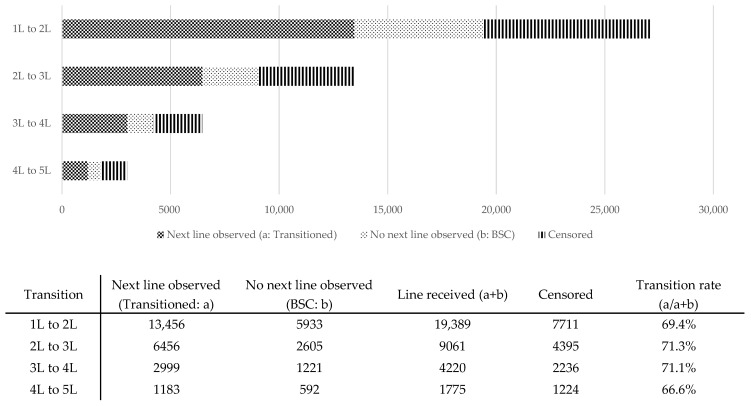
Transition to subsequent line or best supportive care.

Transition rates to subsequent first to fifth treatment lines were relatively consistent across each line, ranging from 66.6% to 71.3% (first to second, 69.4%; second to third, 71.3%; third to fourth, 71.1%; fourth to fifth, 66.6%) (Figure 2).

**Figure 3:**
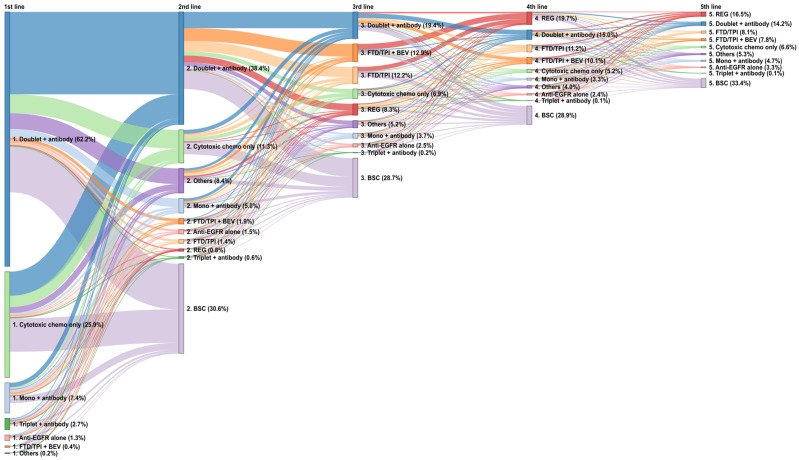
Sequences of treatment regimens by drug class from first to fifth lines in the overall population. Antibody, VEGF inhibitor or anti-EGFR antibody; BEV, bevacizumab; BSC, best supportive care; cytotoxic chemo, cytotoxic chemotherapy; doublet, doublet chemotherapy; EGFR, endothelial growth factor receptor; FTD/TPI, trifluridine/tipiracil; mono, monotherapy; REG, regorafenib; triplet, triplet chemotherapy.

**Figure 4:**
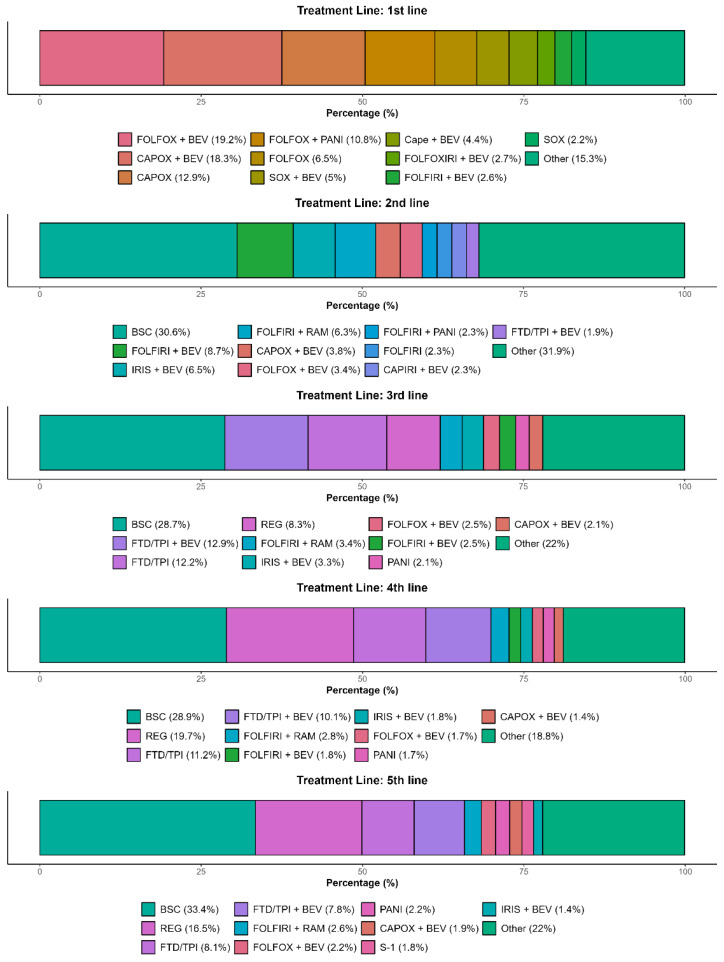
Share of treatment regimens in each treatment line in the overall population. BEV, bevacizumab; BSC, best supportive care; Cape, capecitabine; CAPIRI, capecitabine and irinotecan; CAPOX, capecitabine and oxaliplatin; FOLFIRI, leucovorin, fluorouracil, and irinotecan; FOLFOX, leucovorin, fluorouracil, and oxaliplatin; FOLFOXIRI, leucovorin, fluorouracil, oxaliplatin, and irinotecan; FTD/TPI, trifluridine/tipiracil; IRIS, S-1 and irinotecan; PANI, panitumumab; SOX, S-1 and oxaliplatin; RAM, ramucirumab; REG, regorafenib.

In the overall population, 74.1% of patients started regimens with molecular targeted drugs and more than 60% started with doublet chemotherapy plus an antibody (i.e., VEGF inhibitor or anti-EGFR antibody) regimen (Figure 3). The major doublet chemotherapy and antibody regimens involved leucovorin, fluorouracil, and oxaliplatin (FOLFOX) or oxaliplatin and capecitabine (CAPOX) plus BEV followed by FOLFOX plus anti-EGFR antibody (i.e., PANI or CET), comprising 50.2% of first-line treatments (Figure 4). As a first-to-second-line treatment sequence, 20.1% of patients transitioned from one to another regime of doublet chemotherapy plus antibody treatment, which was the most frequent sequence, and 11.2% of patients transitioned from doublet chemotherapy plus antibody treatment to the BSC (Figure 3). Although 38.4% of patients transitioned to doublet chemotherapy plus an antibody regime as second-line treatment, transition to the BSC was observed as a common treatment pathway (30.6%) (Figure 3).

### 3.2. Patient Characteristics and Early-Line Treatment Sequences in Patients Who Received Second-Line Treatment

In total, 9061 patients received second-line treatment. Patient characteristics at the end of this treatment are presented in Table 1, stratified by those who either transitioned to third-line treatment or the BSC. Overall, the median age was 70.0 years old (IQR: 62–75) and 60.8% of patients were men. Those transitioning to third-line treatment had higher proportions of metastasis and comorbidities and longer median durations of first- and second-line treatments compared to those transitioning to the BSC. Additionally, the patients continuing to third-line treatment were more likely to have received an IRI- and OX-based regimen with VEGF inhibitors and/or anti-EGFR antibodies in first- and second-line treatments.

Comparing patient characteristics between the baseline and the initiation of third-line treatment (6456 patients) indicates that the proportion of metastases and the prevalence of comorbidities had increased by the time of third-line treatment (Table S2). Liver metastases were present in 44.9% of patients at baseline compared to 52.5% at the start of third-line treatment, while the proportion of patients with lung metastases increased from 21.0% to 30.2%. The most common comorbidity, hypertension, was present in 28.4% of patients at baseline and 51.4% at the start of third-line treatment; meanwhile, the prevalence of peripheral neuropathy increased from 6.6% to 26.0%, and that of hand–foot syndrome rose from 12.1% to 38.7%, respectively.

To provide further clarification, the first-to-fifth-line treatment sequences were analyzed, focusing on patients who received second-line treatment (Figure S1). Doublet chemotherapy plus antibody regimens were used for more than half of patients in first- (66.9%) and second-line (56.4%) treatment and were the most common conditions leading to third-line treatment. The proportions of doublet chemotherapy plus antibody treatment in the first and second lines were larger in this population compared with the overall population.

### 3.3. Factors Associated with Continuation to Third-Line Treatment

Factors associated with continuation to third-line treatment, determined using logistic regression analysis, are shown in Table 2. Patients under 65 years of age were more likely to continue to third-line treatment than older patients (OR: 1.50; 95% CI: 1.36–1.67). The duration of first-line treatment was a significant factor; patients with a duration of longer than 180 days were more likely to continue to third-line treatment (OR: 1.24; 95% CI: 1.13–1.37) than those with a shorter duration. Similarly, the duration of second-line treatment was significant; patients with a treatment duration of longer than 120 days were more likely to continue to third-line treatment (OR: 1.70; 95% CI: 1.55–1.86) than those with a shorter duration. Prior therapy with an OX- and IRI-based regimen plus molecular targeted agents was also associated with a higher likelihood of proceeding to third-line treatment (OR: 1.41; 95% CI: 1.27–1.56).

### 3.4. Late-Line Treatments and Sequences in the Overall Population

In the third line, doublet chemotherapy plus an antibody regime was the most common treatment (19.4%), followed by FTD/TPI plus BEV (12.9%), FTD/TPI (12.2%), and REG (8.3%) (Figure 3). Of patients with REG as third-line treatment, 35.6% received an initial dose of 80 mg, 32.8% received 120 mg, 29.3% received 160 mg, 2.0% received 40 mg, and 0.4% received more than 200 mg. As for FTD/TPI, 38.8% of patients received a reduced dose at initial prescription. In fourth-line treatment, many patients transitioned to an REG regimen after receiving FTD/TPI or FTD/TPI plus BEV in the third-line setting, revealing a common treatment sequence from FTD/TPI-based regimens to REG. Although FTD/TPI and REG were common as third-line or later treatments, nearly half of the patients received other regimens.

The median durations of treatment in patients reaching the third line and beyond were 87 days (IQR: 43–175) for third-line, 78 days (IQR: 38–147) for fourth-line, and 71 days (IQR: 35–140) for fifth-line treatment. Regimen-specific durations of major third- to fifth-line treatments are presented in Table 3.

## 4. Discussion

This study revealed real-world treatment sequences of systemic chemotherapy in patients with mCRC, particularly for those who received second- and later-line treatment. After each line, approximately 70% of patients transitioned to subsequent lines, whereas 30% were unable to continue their treatment. Compared with a previous study using the MDV database in Japan, the transition rates reported here were slightly higher. This could be due to different study populations. Our research included broadly defined patients with mCRC, such as the non-MTC cohort; this cohort consisted of patients who were unable to start molecular targeted drug therapy in their first-line treatment, was unique to our analysis, and avoided sample selection bias, as it likely included frail or elderly patients. Another possible explanation could be the study periods; the patients in the previous research started treatment between 2016 and 2019, but the those in the present study mainly started treatment between 2017 and 2022. Two early-stage clinical studies showing the efficacy and safety of FTD/TPI plus BEV as third-line treatment were published in 2017 and 2020 [25,26]. Thus, this new evidence may have influenced daily practice and affected transition rates.

At the end of the second line, patient characteristics were different between patients who continued to third-line treatment and those who transitioned to the BSC. Accordingly, our logistic regression analysis showed several statistically significant factors in continuing to third-line treatment, one of which was the prior use of OX and IRI plus VEGF inhibitors and/or anti-EGFR antibodies. This result corresponds with a previous study, suggesting the importance of using all active key drugs [27]. VEGF inhibitors are known to suppress tumor angiogenesis and reduce intra-tumoral pressure, thereby improving drug penetration [28,29,30]. Similarly, the efficacy of EGFR inhibitors in RAS wild-type CRC is well established [31,32,33]. These biological and clinical mechanisms contribute to prolonged survival and align with our logistic regression findings, which indicate that patients receiving OX/IRI combined with anti-VEGF or anti-EGFR regimens tend to proceed to third-line treatment. Other factors for continuing to the third line included durations of first- and second-line treatments. A higher proportion of patients who continued to the third line tended to have hypertension, peripheral neuropathy, or hand–foot syndrome compared with patients who transitioned to the BSC, indicating potential side effects from longer durations of previous lines of OX, IRI, and VEGF inhibitors and/or anti-EGFR antibodies. It was considered that these side effects were well managed and, consequently, both patients and physicians made the decision to continue to third-line treatment. A similar pattern was observed in those with metastases. Patients with liver and lung metastases frequently have poor prognosis and are often transitioned to the BSC in early treatment; however, they were more likely to transition to third-line treatment in our analysis. We speculate that patients in worse condition are more willing to receive further therapy and require all possible means of treatment. Although their outcomes improve in earlier lines of therapy, they tend to need subsequent treatments more quickly than patients without metastases. Consequently, a higher proportion of these patients reached third-line treatment in our study. Finally, patients under 65 years of age were more likely to receive third-line treatment than older patients, suggesting that older individuals were less likely to continue to later-line settings. Similar trends were observed in a Dutch study, which showed an increased use of the BSC among older patients with mCRC since 2015, owing to increased frailty and concerns about chemotherapy-related toxicity [34]. Considering these findings together suggests that later-line treatment may be challenging for older patients, even though it is crucial from both quality-of-life and OS perspectives. Future research is necessary to investigate more appropriate treatment sequences and management approaches to improve patient outcomes.

Treatment duration was also linked to difficulties in continuing to later-line treatments for patients with mCRC. As recommended in treatment guidelines, REG, FTD/TPI, and FTD/TPI plus BEV regimens have become standard later-line options in Japan over the past 10 years; however, the durations of these regimens are shorter than those observed in randomized trials [35,36,37]. A recent study using real-world data showed that FTD/TPI is associated with higher hematologic toxicity, particularly neutropenia, whereas regorafenib has higher rates of nonhematologic toxicities such as hand–foot syndrome, fatigue, hypertension, and hepatotoxicity [38]. Even when considering the different conditions between randomized trials and real-world practice, this suggests that important unmet medical needs still remain with current standard regimens and more treatment options are necessary.

The treatment landscape for stage IV colorectal cancer is rapidly advancing, with several promising therapies currently in development. Fruquintinib was approved for late-line treatment and an HER2-targeted therapy, tucatinib plus trastuzumab [39], received FDA accelerated approval in early 2023, providing a new treatment option for HER2-positive colorectal cancer patients. Furthermore, multiple drug combinations are being developed to target KRAS G12C mutations. Examples include sotorasib combined with panitumumab and adagrasib combined with cetuximab, which are emerging as promising approaches based on favorable clinical outcomes [40,41,42]. These drugs have been recognized and recommended in international guidelines (e.g., ESMO Living Guideline) [10]. Patients harboring these targeted mutations are expected to transition to newly developed therapies, resulting in treatment sequences that differ from those observed in many individuals in this study. Additionally, advanced immunotherapy combinations, such as zanzalintinib combined with atezolizumab, have demonstrated promising OS outcomes in recent reports [43,44]. These therapies represent a significant step forward in personalized and targeted treatments for mCRC, offering new potential for improved outcomes.

There were several limitations in this study. First, we used claim data, which lacked specific details about treatment regimens and line numbers. Consequently, the algorithms used to define the therapy and sequences in this study were based on several assumptions and may have been partially misclassified. Additionally, the database did not include comprehensive clinical information such as genetic testing results, which are crucial for understanding the clinical profiles of patients with mCRC. Another significant limitation was the difficulty in examining treatment sequence based on RAS statuses, due to the lack of information them. In future research, treatment sequences incorporating genetic information will be necessary to obtain a better understanding of personalized treatment. Furthermore, performance status and genetic mutation information were unavailable to adjust for logistic regression. Therefore, the factors observed in the model should be considered as having associative rather than causal relationships and interpreted with caution. On the other hand, the claim data allowed for the extraction of clinical information such as patients’ metastatic status and complications from diagnostic records, which covered several clinical aspects. Second, the MDV database is hospital-based, meaning that the included information is limited to treatments performed at MDV-registered hospitals. Therefore, patients would be lost to follow-up on transfer to another hospital. Nonetheless, anticancer treatments are generally continued at the initiating medical institution, which supports some level of continuity. Third, the database used in this study included data from about one quarter of Japan’s diagnosis procedure combination (DPC) hospitals (i.e., acute care hospitals). Active treatment—that is, not the BSC—is generally performed in DPC hospitals in Japan; this may have induced selection bias in the study population, and owing to the scope of the database, generalization of the results may be limited to patients in hospitals under similar conditions.

## 5. Conclusions

In this study, we described treatment sequences for patients with mCRC in Japan. In a real-world setting, approximately 70% of patients were able to transition to subsequent treatment lines, but the remaining 30% were unable to continue treatment. The results highlight that appropriate early treatment is crucial for transitioning to later-line treatments. Together, these findings suggest that new treatment options and further research are necessary to meet patients’ needs, especially in later-line settings, in which patients are likely to encounter more obstacles to improving quality of life and survival.

## Figures and Tables

**Table 1:** Patient characteristics at the end of the second-line treatment by next treatment status.

	Transited to Third Line	Transited to BSC	*p* Value ^a^
	(n = 6456)	(n = 2605)	
Age, years, median (IQR)	69 (61, 74)	71 (64, 77)	<0.001
Sex, n (%)			0.638
Male	3937 (61.0%)	1574 (60.4%)	
Female	2519 (39.0%)	1031 (39.6%)	
Index year, n (%)			0.411
2017	1205 (18.7%)	467 (17.9%)	
2018	1284 (19.9%)	557 (21.4%)	
2019	1430 (22.1%)	545 (20.9%)	
2020	1349 (20.9%)	534 (20.5%)	
2021	1006 (15.6%)	420 (16.1%)	
2022	182 (2.8%)	82 (3.1%)	
Primary tumor location, n (%)			0.952
Right side	1810 (28.0%)	729 (28.0%)	
Left side	4340 (67.2%)	1745 (67.0%	
Both	162 (2.5%)	69 (2.6%)	
Unknown	144 (2.2%)	62 (2.4%)	
Metastatic sites, n (%)			
Liver	3302 (51.1%)	1095 (42.0%)	<0.001
Lung	1834 (28.4%)	582 (22.3%)	<0.001
Peritoneal	1088 (16.9%)	422 (16.2%)	<0.001
Lymph node	891 (13.8%)	338 (13.0%)	<0.001
Bone	304 (4.7%)	123 (4.7%)	<0.001
Brain	45 (0.7%)	28 (1.1%)	0.024
Other	292 (4.5%)	123 (4.7%)	<0.001
Comorbidity, n (%)			
Hypertension	3188 (49.4%)	1228 (47.1%)	<0.001
Peripheral neuropathy	1606 (24.9%)	601 (23.1%)	<0.001
Hand–foot syndrome	2390 (37.0%)	841 (32.3%)	<0.001
Anemia	467 (7.2%)	280 (10.7%)	<0.001
Leukopenia	342 (5.3%)	110 (4.2%)	<0.001
Interstitial pneumonitis	110 (1.7%)	59 (2.3%)	0.002
Proteinuria	96 (1.5%)	26 (1.0%)	0.071
Size of hospital, n (%)			<0.001
<200 beds	302 (4.7%)	170 (6.5%)	
200–499 beds	3576 (55.4%)	1470 (56.4%)	
≥500 beds	2578 (39.9%)	965 (37.0%)	
Designated cancer hospital, n (%)	5108 (79.1%)	1974 (75.8%)	<0.001
Treatment duration, median (IQR)			
First line	196 (116, 314)	169 (98, 288)	<0.001
Second line	143 (71, 254)	99 (42, 204)	<0.001
Previous treatment, n (%)			
OX and IRI plus VEGF inhibitors and/or anti-EGFR antibodies	2753 (42.6%)	843 (32.4%)	<0.001

BSC, best supportive care; EGFR, endothelial growth factor receptor; IRI, irinotecan; IQR, interquartile range; OX, oxaliplatin; VEGF, vascular endothelial growth factor. ^a^ Wilcoxon rank-sum test for age and treatment duration, otherwise Pearson’s Chi-squared test.

**Table 2:** Logistic regression analysis of factors associated with continuation to third-line treatment.

		OR	95% CI	*p* Value
Age	<65 years	1.50	(1.36, 1.67)	<0.001
Sex	Female	1.00	(0.91, 1.10)	0.977
Primary tumor location				
Right side		1.07	(0.96, 1.19)	0.200
Both left and right sides		1.06	(0.79, 1.43)	0.711
Unknown		0.98	(0.72, 1.34)	0.893
Treatment duration				
First line	≥180 days	1.24	(1.13, 1.37)	<0.001
Second line	≥120 days	1.70	(1.55, 1.86)	<0.001
Previous treatment				
OX and IRI plus VEGF inhibitors and/or anti-EGFR antibodies		1.41	(1.27, 1.56)	<0.001
Metastatic sites				
Liver		1.42	(1.29, 1.56)	<0.001
Lung		1.14	(1.01, 1.29)	0.031
Peritoneal		1.04	(0.90, 1.20)	0.613
Lymph node *		0.96	(0.83, 1.11)	0.553

CI, confidence interval; EGFR, endothelial growth factor receptor; IRI, irinotecan; OR, odds ratio; OX, oxaliplatin; VEGF, vascular endothelial growth factor. * includes regional lymph nodes.

**Table 3:** Duration of major treatment regimens in third- and later-line treatments.

**Treatment Lines and Regimens**	**n**	**Median**	**IQR**
		**(Days)**	**(Days)**
Third-line	4220	87	(43–175)
FTD/TPI	682	65	(38–101)
FTD/TPI + EV	708	92	(50–173)
REG	446	49	(21–98)
Fourth-line	1775	78	(38–147)
FTD/TPI	282	66	(38–102.75)
FTD/TPI + BEV	247	108	(71–192)
REG	434	49	(21–84)
Fifth-line	713	71	(35–140)
FTD/TPI	85	60	(23–94)
FTD/TPI + BEV	78	96.5	(50–179)
REG	164	49	(21–98)

BEV, bevacizumab; FTD/TPI, trifluridine/tipiracil; IQR, interquartile range; REG, regorafenib.

## Data Availability

The data sets generated during and/or analyzed during the current study are not publicly available due to research contracts with the data suppliers.

## Supplementary Materials

The following supporting information can be downloaded at https://www.mdpi.com/article/10.3390/cancers17243962/s1. Table S1: Patient characteristics on index date in the overall population; Table S2: Characteristics at start of first-line and third-line treatment of patients who started third-line treatment; Figure S1: Sequences of treatment regimens by drug class from first to fifth lines in patients who received second-line treatment.

## References

[1] Sung H., Ferlay J., Siegel R.L., Laversanne M., Soerjomataram I., Jemal A., Bray F. (2021). Global Cancer Statistics 2020: GLOBOCAN Estimates of Incidence and Mortality Worldwide for 36 Cancers in 185 Countries. CA Cancer J. Clin..

[2] Biller L.H., Schrag D. (2021). Diagnosis and Treatment of Metastatic Colorectal Cancer: A Review. JAMA.

[3] Dasari A., Lonardi S., Garcia-Carbonero R., Elez E., Yoshino T., Sobrero A., Yao J., García-Alfonso P., Kocsis J., Cubillo Gracian A. (2023). Fruquintinib versus Placebo in Patients with Refractory Metastatic Colorectal Cancer (FRESCO-2): An International, Multicentre, Randomised, Double-Blind, Phase 3 Study. Lancet.

[4] Shitara K., Muro K., Watanabe J., Yamazaki K., Ohori H., Shiozawa M., Takashima A., Yokota M., Makiyama A., Akazawa N. (2024). Baseline CtDNA Gene Alterations as a Biomarker of Survival after Panitumumab and Chemotherapy in Metastatic Colorectal Cancer. Nat. Med..

[5] Yamada Y., Takahari D., Matsumoto H., Baba H., Nakamura M., Yoshida K., Yoshida M., Iwamoto S., Shimada K., Komatsu Y. (2013). Leucovorin, Fluorouracil, and Oxaliplatin plus Bevacizumab versus S-1 and Oxaliplatin plus Bevacizumab in Patients with Metastatic Colorectal Cancer (SOFT): An Open-Label, Non-Inferiority, Randomised Phase 3 Trial. Lancet Oncol..

[6] Yamazaki K., Nagase M., Tamagawa H., Ueda S., Tamura T., Murata K., Eguchi Nakajima T., Baba E., Tsuda M., Moriwaki T. (2016). Randomized Phase III Study of Bevacizumab plus FOLFIRI and Bevacizumab plus MFOLFOX6 as First-Line Treatment for Patients with Metastatic Colorectal Cancer (WJOG4407G). Ann. Oncol..

[7] Loupakis F., Cremolini C., Masi G., Lonardi S., Zagonel V., Salvatore L., Cortesi E., Tomasello G., Ronzoni M., Spadi R. (2014). Initial Therapy with FOLFOXIRI and Bevacizumab for Metastatic Colorectal Cancer. N. Engl. J. Med..

[8] Deng Y., Chi P., Lan P., Wang L., Chen W., Cui L., Chen D., Cao J., Wei H., Peng X. (2019). Neoadjuvant Modified FOLFOX6 with or without Radiation versus Fluorouracil plus Radiation for Locally Advanced Rectal Cancer: Final Results of the Chinese FOWARC Trial. J. Clin. Oncol..

[9] Schnipper L.E., Davidson N.E., Wollins D.S., Tyne C., Blayney D.W., Blum D. (2015). American Society of Clinical Oncology Statement: A Conceptual Framework to Assess the Value of Cancer Treatment Options. J. Clin. Oncol..

[10] Cervantes A., Adam R., Roselló S., Arnold D., Normanno N., Taïeb J., Seligmann J., De Baere T., Osterlund P., Yoshino T. (2023). Metastatic Colorectal Cancer: ESMO Clinical Practice Guideline for Diagnosis, Treatment and Follow-Up☆. Ann. Oncol..

[11] JSCCR Guidelines 2022 for the Treatment of Colorectal Cancer. https://www.jsccr.jp/guideline/2022/index_guide.html.

[12] Colorectal Cancer Facts & Figures. https://www.cancer.org/research/cancer-facts-statistics/colorectal-cancer-facts-figures.html.

[13] Shinozaki E., Makiyama A., Kagawa Y., Satake H., Tanizawa Y., Cai Z., Piao Y. (2021). Treatment Sequences of Patients with Advanced Colorectal Cancer and Use of Second-Line FOLFIRI with Antiangiogenic Drugs in Japan: A Retrospective Observational Study Using an Administrative Database. PLoS ONE.

[14] Satake H., Kagawa Y., Shinozaki E., Tanizawa Y., Jin L., Cai Z., Makiyama A. (2022). Real-World Data Analysis of Second-Line Antiangiogenic Targeted Treatments Following Anti-Epidermal Growth Factor Receptor Monoclonal Antibodies and First-Line FOLFOX for Patients with Metastatic Colorectal Cancer. Adv. Ther..

[15] Yamazaki K., Yuki S., Oki E., Sano F., Makishima M., Aoki K., Hamano T., Yamanaka T. (2021). Real-World Evidence on Second-Line Treatment of Metastatic Colorectal Cancer Using Fluoropyrimidine, Irinotecan, and Angiogenesis Inhibitor. Clin. Color. Cancer.

[16] Teng C.-L.J., Wang C.-Y., Chen Y.-H., Lin C.-H., Hwang W.-L. (2015). Optimal Sequence of Irinotecan and Oxaliplatin-Based Regimens in Metastatic Colorectal Cancer: A Population-Based Observational Study. PLoS ONE.

[17] Kagawa Y., Wang C., Piao Y., Jin L., Tanizawa Y., Cai Z., Sunakawa Y. (2024). Real-World Evidence of FOLFIRI Combined with Anti-Angiogenesis Inhibitors or Anti-EGFR Antibodies for Patients with Early Recurrence Colorectal Cancer after Adjuvant FOLFOX/CAPOX Therapy: A Japanese Claims Database Study. Target. Oncol..

[18] Kagawa Y., Shinozaki E., Okude R., Tone T., Kunitomi Y., Nakashima M. (2023). Real-World Evidence of Trifluridine/Tipiracil plus Bevacizumab in Metastatic Colorectal Cancer Using an Administrative Claims Database in Japan. ESMO Open.

[19] Nakashima M., Takeuchi M., Kawakami K. (2020). Effectiveness and Safety of Regorafenib vs. Trifluridine/Tipiracil in Unresectable Colorectal Cancer: A Retrospective Cohort Study. Clin. Color. Cancer.

[20] Roset M., Amonkar M., Patel R., Lara N., Kothari S. (2022). Real-World Treatment Patterns and Clinical Outcomes for Standard of Care Regimens in Patients with Deficient MMR or MSI-High Metastatic Colorectal and Non-Colorectal Cancer: A Retrospective Chart Review Study in France. Adv. Ther..

[21] Min S.T., Roohullah A., Tognela A., Jalali A., Lee M., Wong R., Shapiro J., Burge M., Yip D., Nott L. (2022). Patient Demographics and Management Landscape of Metastatic Colorectal Cancer in the Third-Line Setting: Real-World Data in an Australian Population. Asia Pac. J. Clin. Oncol..

[22] MDV Database Overview. https://en.mdv.co.jp/ebm/about-mdv-database/mdv-database-overview/.

[23] Laurent T., Simeone J., Kuwatsuru R., Hirano T., Graham S., Wakabayashi R., Phillips R., Isomura T. (2022). Context and Considerations for Use of Two Japanese Real-World Databases in Japan: Medical Data Vision and Japanese Medical Data Center. Drugs Real World Outcomes.

[24] Hess L.M., Li X., Wu Y., Goodloe R.J., Cui Z.L. (2021). Defining Treatment Regimens and Lines of Therapy Using Real-World Data in Oncology. Future Oncol..

[25] Kuboki Y., Nishina T., Shinozaki E., Yamazaki K., Shitara K., Okamoto W., Kajiwara T., Matsumoto T., Tsushima T., Mochizuki N. (2017). TAS-102 plus Bevacizumab for Patients with Metastatic Colorectal Cancer Refractory to Standard Therapies (C-TASK FORCE): An Investigator-Initiated, Open-Label, Single-Arm, Multicentre, Phase 1/2 Study. Lancet Oncol..

[26] Pfeiffer P., Yilmaz M., Möller S., Zitnjak D., Krogh M., Petersen L.N., Poulsen L.Ø., Winther S.B., Thomsen K.G., Qvortrup C. (2020). TAS-102 with or without Bevacizumab in Patients with Chemorefractory Metastatic Colorectal Cancer: An Investigator-Initiated, Open-Label, Randomised, Phase 2 Trial. Lancet Oncol..

[27] Grothey A., Sargent D. (2005). Overall Survival of Patients With Advanced Colorectal Cancer Correlates With Availability of Fluorouracil, Irinotecan, and Oxaliplatin Regardless of Whether Doublet or Single-Agent Therapy Is Used First Line. J. Clin. Oncol..

[28] Jain R.K. (2001). Normalizing Tumor Vasculature with Anti-Angiogenic Therapy: A New Paradigm for Combination Therapy. Nat. Med..

[29] Jain R.K. (2005). Normalization of Tumor Vasculature: An Emerging Concept in Antiangiogenic Therapy. Science.

[30] Patel S.A., Nilsson M.B., Le X., Cascone T., Jain R.K., Heymach J.V. (2023). Molecular Mechanisms and Future Implications of VEGF/VEGFR in Cancer Therapy. Clin. Cancer Res..

[31] Watanabe J., Muro K., Shitara K., Yamazaki K., Shiozawa M., Ohori H., Takashima A., Yokota M., Makiyama A., Akazawa N. (2023). Panitumumab vs Bevacizumab Added to Standard First-Line Chemotherapy and Overall Survival among Patients with RAS Wild-Type, Left-Sided Metastatic Colorectal Cancer: A Randomized Clinical Trial. JAMA.

[32] Heinemann V., von Weikersthal L.F., Decker T., Kiani A., Vehling-Kaiser U., Al-Batran S.-E., Heintges T., Lerchenmüller C., Kahl C., Seipelt G. (2014). FOLFIRI plus Cetuximab versus FOLFIRI plus Bevacizumab as First-Line Treatment for Patients with Metastatic Colorectal Cancer (FIRE-3): A Randomised, Open-Label, Phase 3 Trial. Lancet Oncol..

[33] Venook A.P., Niedzwiecki D., Lenz H.-J., Innocenti F., Fruth B., Meyerhardt J.A., Schrag D., Greene C., O’Neil B.H., Atkins J.N. (2017). Effect of First-Line Chemotherapy Combined with Cetuximab or Bevacizumab on Overall Survival in Patients with KRAS Wild-Type Advanced or Metastatic Colorectal Cancer: A Randomized Clinical Trial. JAMA.

[34] Baltussen J.C., de Glas N.A., Liefers G.-J., Slingerland M., Speetjens F.M., van den Bos F., Cloos-van Balen M., Verschoor A.J., Jochems A., Spierings L.E.A.M.M. (2023). Time Trends in Treatment Patterns and Survival of Older Patients with Synchronous Metastatic Colorectal Cancer in the Netherlands: A Population-Based Study. Int. J. Cancer.

[35] Li J., Qin S., Xu R., Yau T.C.C., Ma B., Pan H., Xu J., Bai Y., Chi Y., Wang L. (2015). Regorafenib plus Best Supportive Care versus Placebo plus Best Supportive Care in Asian Patients with Previously Treated Metastatic Colorectal Cancer (CONCUR): A Randomised, Double-Blind, Placebo-Controlled, Phase 3 Trial. Lancet Oncol..

[36] Grothey A., Van Cutsem E., Sobrero A., Siena S., Falcone A., Ychou M., Humblet Y., Bouché O., Mineur L., Barone C. (2013). Regorafenib Monotherapy for Previously Treated Metastatic Colorectal Cancer (CORRECT): An International, Multicentre, Randomised, Placebo-Controlled, Phase 3 Trial. Lancet.

[37] Prager G.W., Taieb J., Fakih M., Ciardiello F., Van Cutsem E., Elez E., Cruz F.M., Wyrwicz L., Stroyakovskiy D., Pápai Z. (2023). Trifluridine-Tipiracil and Bevacizumab in Refractory Metastatic Colorectal Cancer. N. Engl. J. Med..

[38] Nevala-Plagemann C., Sama S., Ying J., Shen J., Haaland B., Florou V., Garrido-Laguna I. (2023). A Real-World Comparison of Regorafenib and Trifluridine/Tipiracil in Refractory Metastatic Colorectal Cancer in the United States. J. Natl. Compr. Canc. Netw..

[39] Strickler J.H., Cercek A., Siena S., André T., Ng K., Van Cutsem E., Wu C., Paulson A.S., Hubbard J.M., Coveler A.L. (2023). Tucatinib plus Trastuzumab for Chemotherapy-Refractory, HER2-Positive, RAS Wild-Type Unresectable or Metastatic Colorectal Cancer (MOUNTAINEER): A Multicentre, Open-Label, Phase 2 Study. Lancet Oncol..

[40] Fakih M.G., Salvatore L., Esaki T., Modest D.P., Lopez-Bravo D.P., Taieb J., Karamouzis M.V., Ruiz-Garcia E., Kim T.-W., Kuboki Y. (2023). Sotorasib plus Panitumumab in Refractory Colorectal Cancer with Mutated KRAS G12C. N. Engl. J. Med..

[41] Yaeger R., Weiss J., Pelster M.S., Spira A.I., Barve M., Ou S.-H.I., Leal T.A., Bekaii-Saab T.S., Paweletz C.P., Heavey G.A. (2023). Adagrasib with or without Cetuximab in Colorectal Cancer with Mutated KRAS G12C. N. Engl. J. Med..

[42] Pietrantonio F., Salvatore L., Esaki T., Modest D.P., Lopez-Bravo D.P., Taieb J., Karamouzis M.V., Ruiz-Garcia E., Kim T.W., Kuboki Y. (2025). Overall Survival Analysis of the Phase III CodeBreaK 300 Study of Sotorasib plus Panitumumab versus Investigator’s Choice in Chemorefractory KRAS G12C Colorectal Cancer. J. Clin. Oncol..

[43] Tabernero J., Saeed A., Parikh A., Wang Y., Wang Z., Schwickart M., Curran D., Hecht J. (2023). P-35 Zanzalintinib (XL092) in Combination with Atezolizumab for Previously Treated Metastatic Colorectal Cancer. Ann. Oncol..

[44] Hecht J.R., Park Y.S., Tabernero J., Lee M.-A., Lee S., Virgili A.C., Van den Eynde M., Fontana E., Fakih M., Asghari G. (2025). Zanzalintinib plus Atezolizumab versus Regorafenib in Refractory Colorectal Cancer (STELLAR-303): A Randomised, Open-Label, Phase 3 Trial. Lancet.

