# Supplementary material for: Treatment Sequences in Patients with Metastatic Colorectal Cancer in Japan: Real-World Evidence of First- to Fifth-Line Treatments
Source: Cancers (Basel). 2025 Dec 12;17(24):3962. doi: 10.3390/cancers17243962 (PMC12731131; doi:10.3390/cancers17243962)
Supplement: Supplementary file 1 [file cancers-17-03962-s001.zip › cancers-3975575-supplementary.pdf]

Supplementary Information

Title: Treatment Sequences in Patients with Metastatic Colorectal Cancer in Japan: Real-World Evidence of First- to Fifth-Line Treatments

Yoshinori Kagawa[1,] Tsuyoshi Osaka[2], Toshiki Imamura[2], Hiroyo Kuwabara[2]

[1]Department of Gastroenterological Surgery, Osaka International Cancer Institute, Osaka, Japan

[2]Medical Affairs, Japan Oncology Business Unit, Takeda Pharmaceutical Company Limited, Tokyo, Japan

List of supplementary information

Table S1	Patient characteristics on index date in the overall population
Table S2	Characteristics at start of first-line and third-line treatment of patients who started third-line treatment
Figure S1	Sequences of treatment regimens by drug class from first to fifth lines in patients who received second-line treatment

Table S1 Patient characteristics on index date in the overall population.

	ERC<br>(n = 1335)	MTC<br>(n = 20093)	Non-MTC<br>(n = 5672)
Age, years, median (IQR)	69 (61, 75)	69 (62, 75)	69 (60, 74)
Age ≥ 65 years, n (%)	892 (66.8%)	13,625 (67.8%)	3660 (64.5%)
Sex, n (%)			
Male	785 (58.8%)	12,232 (60.9%)	3605 (63.6%)
Female	550 (41.2%)	7861 (39.1%)	2067 (36.4%)
Index year, n (%)			
2017	216 (16.2%)	2474 (12.3%)	643 (11.3%)
2018	246 (18.4%)	2747 (13.7%)	774 (13.6%)
2019	249 (18.7%)	3248 (16.2%)	855 (15.1%)
2020	264 (19.8%)	3497 (17.4%)	1002 (17.7%)
2021	261 (19.6%)	3924 (19.5%)	1149 (20.3%)
2022	99 (7.4%)	4203 (20.9%)	1249 (22.0%)
Primary tumor location, n (%)			
Right side	473 (35.4%)	5701 (28.4%)	1335 (23.5%)
Left side	811 (60.7%)	13,384 (66.6%)	3968 (70.0%)
Both	44 (3.3%)	503 (2.5%)	122 (2.2%)
Unknown	7 (0.5%)	505 (2.5%)	247 (4.4%)
Metastatic sites, n (%)			
Liver	178 (13.3%)	8867 (44.1%)	1727 (30.4%)
Lung	44 (3.3%)	4083 (20.3%)	762 (13.4%)
Peritoneal	41 (3.1%)	2738 (13.6%)	541 (9.5%)
Lymph node	76 (5.7%)	2477 (12.3%)	533 (9.4%)
Bone	4 (0.3%)	571 (2.8%)	109 (1.9%)
Brain	1 (0.1%)	120 (0.6%)	35 (0.6%)
Other	19 (1.4%)	725 (3.6%)	179 (3.2%)
Comorbidity, n (%)			
Hypertension	375 (28.1%)	6152 (30.6%)	1607 (28.3%)
Peripheral neuropathy	24 (1.8%)	1516 (7.5%)	381 (6.7%)
Hand-foot syndrome	24 (1.8%)	3061 (15.2%)	645 (11.4%)
Anemia	154 (11.5%)	1530 (7.6%)	450 (7.9%)
Leukopenia	1 (0.1%)	432 (2.2%)	87 (1.5%)
Interstitial pneumonitis	3 (0.2%)	151 (0.8%)	33 (0.6%)
Proteinuria	2 (0.1%)	39 (0.2%)	10 (0.2%)
Size of hospital, n (%)			
< 200 beds	88 (6.6%)	1043 (5.2%)	320 (5.6%)
200–499 beds	761 (57.0%)	11,074 (55.1%)	3057 (53.9%)
≥ 500 beds	486 (36.4%)	7976 (39.7%)	2295 (40.5%)
Designated cancer hospital, n (%)	999 (74.8%)	15679 (78.0%)	4476 (78.9%)

CRC, colorectal cancer; ERC, early recurrence cohort; IQR, interquartile range; MTC, molecular targeted therapy cohort; RAS, rat sarcoma viral oncogene homolog.

Table S2 Characteristics at start of first-line and third-line treatment of patients who started third-line treatment.

	Start of first-line treatment (n = 6456)	Start of third-line treatment (n = 6456)
Age, years, median (IQR)	68 (59, 73)	69 (61, 75)
Gender, n (%)		
Male	3937 (61.0%)	–
Female	2519 (39.0%)	–
Year at the start of third-line treatment, n (%)		
2017	1205 (18.7%)	91 (1.4%)
2018	1284 (19.9%)	586 (9.1%)
2019	1430 (22.1%)	977 (15.1%)
2020	1349 (20.9%)	1368 (21.2%)
2021	1006 (15.6%)	1549 (24.0%)
2022	182 (2.8%)	1668 (25.8%)
2023	–	217 (3.4%)
Primary tumor location, n (%)		
Right side	1810 (28.0%)	–
Left side	4340 (67.2%)	–
Both	162 (2.5%)	–
Unknown	144 (2.2%)	–
Metastatic sites, n (%)		
Liver	2899 (44.9%)	3389 (52.5%)
Lung	1353 (21.0%)	1948 (30.2%)
Peritoneal	777 (12.0%)	1157 (17.9%)
Lymph node	742 (11.5%)	935 (14.5%)
Bone	114 (1.8%)	342 (5.3%)
Brain	23 (0.4%)	56 (0.9%)
Other	195 (3.0%)	341 (5.3%)
Comorbidity, n (%)		
Hypertension	1833 (28.4%)	3319 (51.4%)
Peripheral neuropathy	429 (6.6%)	1679 (26.0%)
Hand-foot syndrome	784 (12.1%)	2501 (38.7%)
Anemia	451 (7.0%)	510 (7.9%)
Leukopenia	139 (2.2%)	350 (5.4%)
Interstitial pneumonitis	34 (0.5%)	129 (2.0%)
Proteinuria	11 (0.2%)	99 (1.5%)
Size of hospital, n (%)		
< 200 beds	302 (4.7%)	–
200–499 beds	3576 (55.4%)	–
≥ 500 beds	2578 (39.9%)	–
Designated cancer hospital, n (%)	5108 (79.1%)	–

CRC, colorectal cancer; ERC, early recurrence cohort; IQR, interquartile range; MTC, molecular targeted therapy cohort; RAS, rat sarcoma viral oncogene homolog.

Figure S1. Sequences of treatment regimens by drug class from first to fifth lines in patients who received second-line treatment.

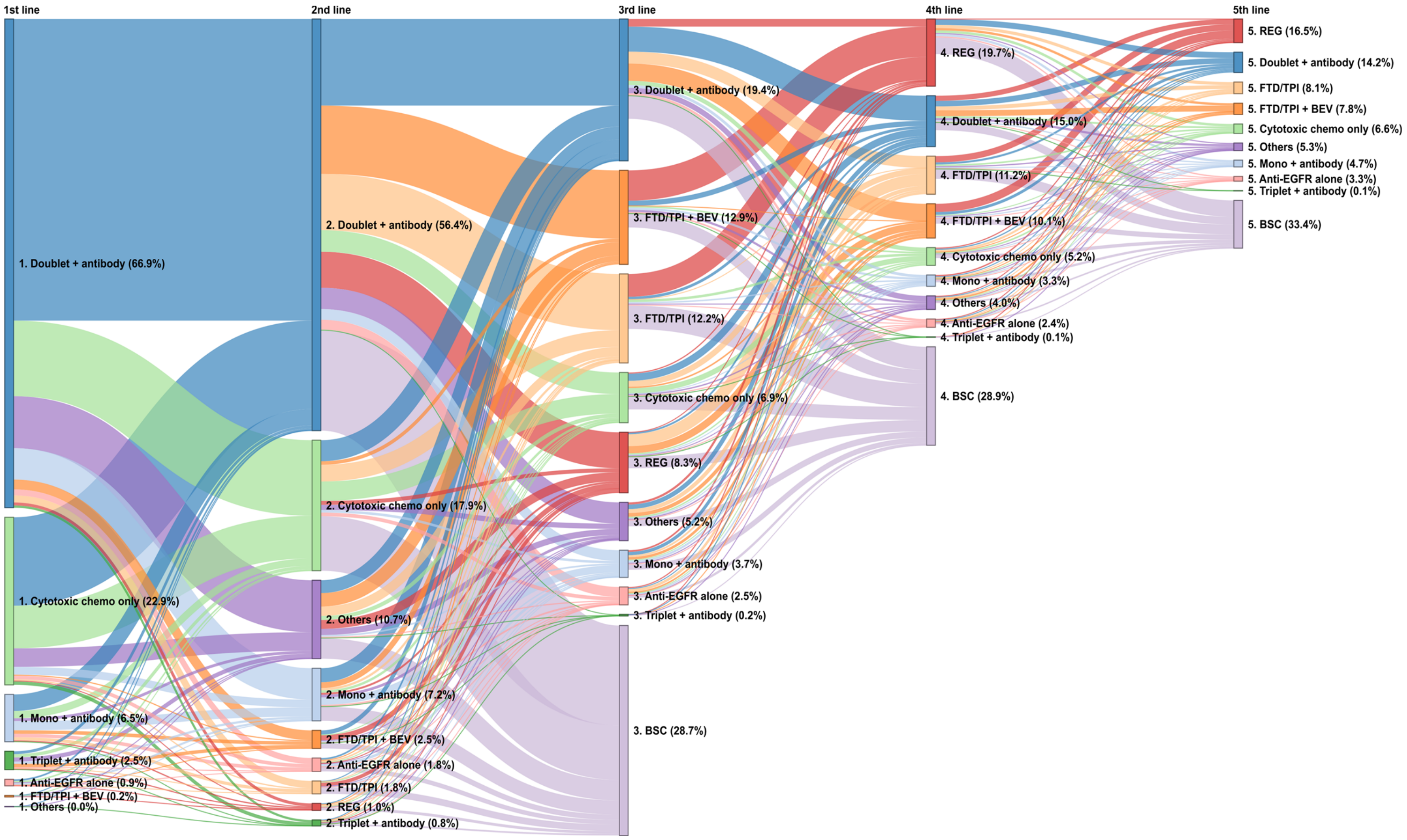

Antibody, VEGF inhibitor or anti-EGFR antibody; BEV, bevacizumab; BSC, best supportive care; cytotoxic chemo, cytotoxic chemotherapy; doublet, doublet chemotherapy; EGFR, endothelial growth factor receptor; FTD/TPI, trifluridine/tipiracil; mono, monotherapy; REG, regorafenib; triplet, triplet chemotherapy.
